# Integrated bioinformatics analysis of key genes involved in progress of colon cancer

**DOI:** 10.1002/mgg3.588

**Published:** 2019-02-11

**Authors:** Haojie Yang, Jiong Wu, Jingjing Zhang, Zhigang Yang, Wei Jin, Ying Li, Lei Jin, Lu Yin, Hua Liu, Zhenyi Wang

**Affiliations:** ^1^ Department of colo‐proctology Yueyang Hospital of Integratd Traditional Chinese and Western Medicine, Shanghai University of Traditional Chinese Medicine Shanghai China; ^2^ Shanghai Changning Maternity & Infant Health Hospital Shanghai China

**Keywords:** bioinformatic analysis, colon cancer, GEO, TCGA

## Abstract

**Background:**

Colon cancer is one of most malignant cancers around worldwide. Nearly 20% patients were diagnosed at colon cancer with metastasis. However, the lack of understanding regarding its pathogenesis brings difficulties to study it.

**Methods:**

In this study, we acquired high‐sequence data from GEO dataset, and performed integrated bioinformatic analysis including differently expressed genes, gene ontology and Kyoto Encyclopedia of Genes and Genomes pathways analysis, protein–protein analysis, survival analysis to analyze the development of colon cancer.

**Results:**

By comparing the colon cancer tissues with normal colon tissues, 109 genes were dysregulated; among them, 83 genes were downregulated and 26 genes were upregulated. Two clusters were founded based on the STRING database and MCODE plugin of cytoscape software. Then, six genes with prognostic value were filtered out in UALCAN website.

**Conclusion:**

We found that *SPP1*, *VIP*, *COL11A1*, *CA2*, *ADAM12*, *INHBA* could provide great significant prognostic value for colon cancer.

## 1 INTRODUCTION

Colon cancer, one of the most malignant cancers around worldwide, has caused more than 50,000 deaths per year (Haggar & Boushey, 2009). Due to the characters of colon cancer, such as incidence hidden, progression rapidly, prone to resistant to chemotherapy (He et al., 2017; Marin et al., 2012), it has brought seriously social and medical burden which arose public concern.

Although large‐scale studies have been carried on to investigate the early diagnosis biomarkers and the mechanism of colon cancer, it is easy for us to be lost in the dense fog when treating colon cancer. Giving to the contribution of the second‐generation gene sequencing (Kamps et al., 2017), it is much helpful for us to uncover the causes and pathogenesis of colon cancer as well as identifying novel biomarkers with great prognostic value.

In this study, we perform integrated analysis including differently expressed genes, gene ontology (GO) analysis, KEGG pathway analysis, survival analysis both to identify a panel of key candidate genes involved in colon cancer, and we found that *SPP1*, *VIP*, *COL11A1*, *CA2*, *ADAM12*, and *INHBA* could provide great significant prognostic value for colon cancer.

## 2 MATERIALS AND METHODS

### 2.1 Ethical compliance

The clinical information and sequence data were acquired according to the requirements of GEO and TCGA databanks. Thus, no ethics committee approval or consent procedure was needed.

### 2.2 Data source

High‐sequence data of GSE62932 (GPL570, Affymetrix Human Genome U133 Plus 2.0 Array) were collected from GEO dataset, which includes 68 colon tissues until 03 June 2018. As GEO is a publicly available dataset, no ethics approval is required. The samples were divided into two groups based on the sample type, a total of 64 colon cancer tissues and 4 normal colon tissues were utilized for the following analysis.

### 2.3 Differently expressed genes in colon cancer

Prior to analyzing the DEGs (differently expressed genes) in colon cancer, the sequence data were normalized using RMA (Robust Multichip Average). Then, we performed DEGs analysis using limma package with the cutoff of *p*‐value <0.05 and |logFC| ≥ 2 (Robinson, McCarthy, & Smyth, 2010). The heatmap was shown by pheatmap R package based on the expression value of DEGs. To better understand how the DEGs involved in the biological process and the signal transduction process, the clusterprofiler R package was carried on the GO and Kyoto Encyclopedia of Genes and Genomes (KEGG) pathway analysis (Yu, Wang, Han, & He, 2012), a *p*‐value <0.05 was considered significant.

### 2.4 Protein–protein network analysis

As genes were interacting with each other, to deep excavate the central genes, STRING database was applied to construct the interaction network of genes (Szklarczyk et al., 2017). Cytoscape software was performed to visualize the relationship between genes (Shannon et al., 2003). For the sake of further research, following the protein–protein network analysis, the MCODE plugin was performed to re‐analyze the clusters among the network according to the k‐core = 2.

### 2.5 Survival analysis to screen the candidate genes

Survival analysis was carried on the UALCAN website (Chandrashekar et al., 2017), which is a portal for survival analysis according to the TCGA dataset. The colon cancer samples were divided into two groups according to gene expression: high expression (with Transcripts per million [TPM] values higher median) and low/median expression (with TPM values lower median). Then, we used the Kaplan–Meier method to analyze the candidate genes of significantly prognostic value with a *p*‐value <0.05.

### 2.6 Validation in TCGA and The Human Protein Atlas

For validation, the candidate genes were assessed both from RNA expression level and protein level by TCGA data portal and The Human Protein Atlas database, respectively. The GEPIA website was applied to exhibit the relative RNA expression level between colon cancer and normal colon tissues while The Human Protein Atlas database was performed to map the protein in the tissues (Tang et al., 2017; Uhlén et al., 2015).

## 3 RESULTS

### 3.1 Differently expressed genes involved in colon cancer

**Figure 1:**
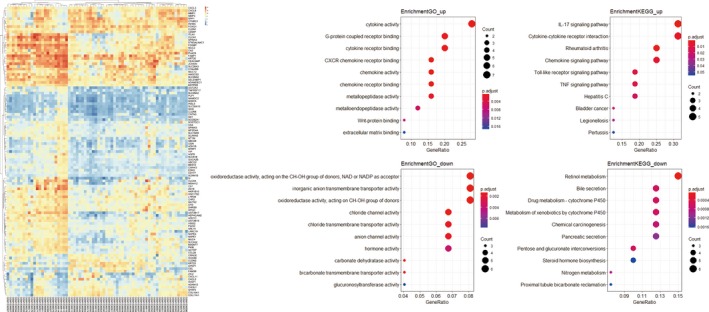
The dysregulated genes involved in colon cancer. Onthe left is the heatmap of dysregulated genes. Onthe middle is the GO analysis of the upregulated and downregulated genes. On the right is the Kyoto Encyclopedia of Genes and Genomes (KEGG) analysis of the upregulated and downregulated genes

In this part, samples were first grouped based on the pathology of colon tissues as colon cancer tissues and normal colon tissues, respectively. Differently expressed genes analysis was performed in succession with the *p*‐value <0.05 and |logFC| ≥ 2. One hundred and nine genes were dysregulated, among them, 83 genes were downregulated and 26 genes were upregulated. To further evaluate the genes’ function, we performed GO analysis and Kyoto Encyclopedia of Genes and Genomes (KEGG) analysis. The upregulated genes are mainly enriched in C‐X‐C chemokine receptor（CXCR) binding, cytokine activity, chemokine activity, chemokine receptor binding, G‐protein coupled receptor binding, IL‐17 signaling pathway, rheumatoid arthritis, cytokine‐cytokine receptor interaction, chemokine signaling pathway, Toll‐like receptor signaling pathway. The downregulated genes were mostly enriched in oxidoreductase activity, acting on the CH–OH group of donors, nicotinamide adenine dinucleotide (NAD) or nicotinamide adenine dinucleotide phosphate (NADP) as acceptor, carbonate dehydratase activity, chloride channel activity, inorganic anion transmembrane transporter activity, oxidoreductase activity, acting on CH–OH group of donors, retinol metabolism, pentose and glucuronate interconversions, bile secretion, drug metabolism ‐ cytochrome P450, metabolism of xenobiotics by cytochrome P450 (Figure 1).

### 3.2 Protein–protein network analysis

**Figure 2:**
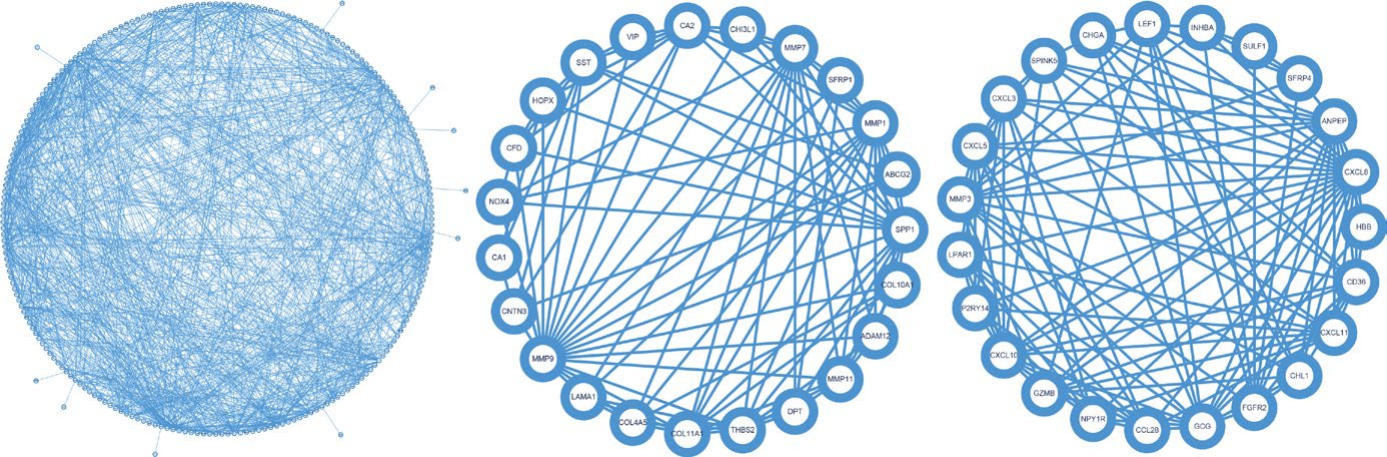
Protein–protein network of dysregulated genes. On the left is the whole interactions between dysregulated genes. On the middle is the cluster1 involved in. On the right is the cluster2 involved in

To deep excavate the key genes involved in the development of colon cancer, we take STRING website to estimate the interaction relationship between genes. One thousand three hundred and twenty‐six pairs involved with 229 proteins were constructed in Cytoscape software (Figure 2). We then utilized MCODE plugin to find densely connected regions based on topology and two dense clusters were discovered. Cluster 1 involved 23 genes and 113 connections (*SULF1*, *NPY1R*, *CXCL8*, *FGFR2*, *CXCL5*, *LEF1*, *CCL28*, *HBB*, *CXCL3*, *CXCL10*, *MMP3*, *CHL1*, *INHBA*, *GCG*, *LPAR1*, *CHGA*, *CD36*, *SPINK5*, *SFRP4*, *ANPEP*, *GZMB*, *CXCL11*, *P2RY14*). Cluster 2 involved 23 genes and 94 connections(*HOPX*, *SPP1*, *NOX4*, *DPT*, *COL10A1*, *MMP11*, *ADAM12*, *LAMA1*, *COL11A1*, *SFRP1*, *MMP1*, *CA2*, *SST*, *CA1*, *THBS2*, *CFD*, *MMP9*, *VIP*, *ABCG2*, *CHI3L1*, *MMP7*, *COL4A5*, *CNTN3*). The density of our protein‐protein network was confirmed with the high degree of nodes, suggesting common competitions for colon cancer.

### 3.3 Survival analysis of key genes

**Figure 3:**
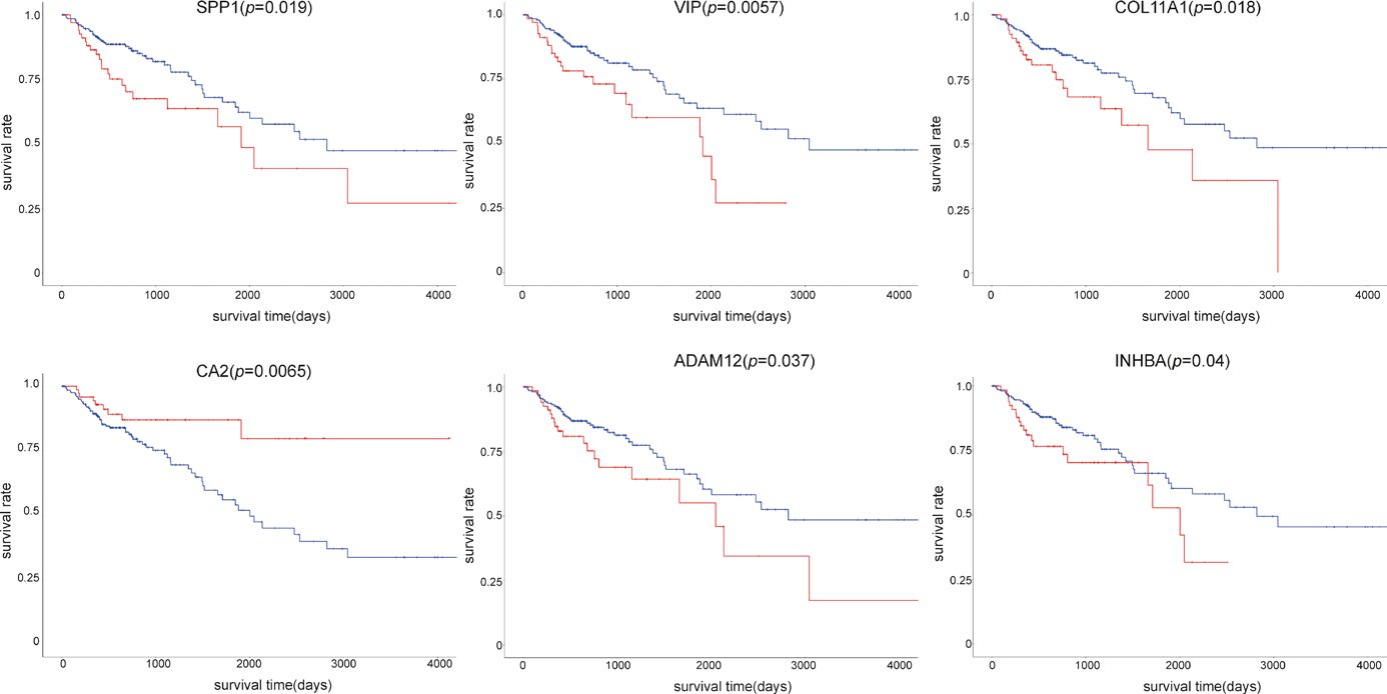
Survival analysis of key genes. The red plots present the high expression of each individuals while the blue plots present the median/low expression of each individuals. SPP1: NC_000004.12; VIP: NC_000006.12; COL11A1: NC_000001.11; CA2: NC_000008.11; ADAM12: NC_000010.11; INHBA: NC_000007.14

To seek the candidate genes which may influence the survival outcome, we perform the survival analysis on the key genes. A total of six candidate genes were screened and were found to have impact on overall survival days, which are *SPP1*, *VIP*, *COL11A1*, *CA2*, *ADAM12*, *INHBA*, respectively (Figure 3). Patients whose tissues have a higher expression of *SPP1*, *VIP*, *COL11A1*, *ADAM12*, *INHBA* had significantly shorter overall survival compared to those with lower expression, while patients with higher expression of *CA2 *have a better prognosis.

### 3.4 Validation in TCGA and the Human Protein Atlas

**Figure 4:**
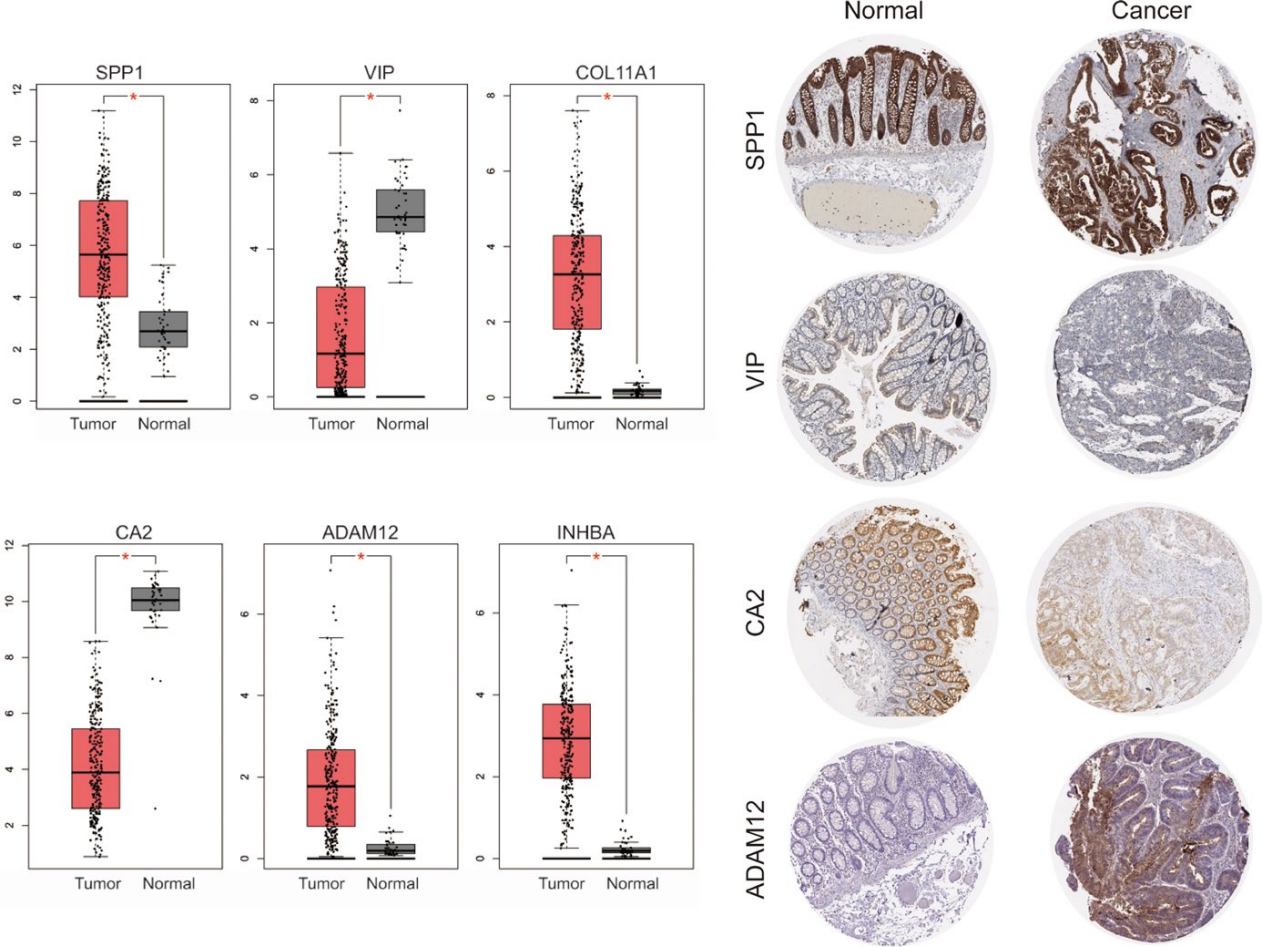
Validation of key genes in TCGA dataset and the human protein Atlas dataset

The RNA expression levels of *SPP1*, *VIP*, *COL11A1*, *CA2*, *ADAM12*, and *INHBA* were validated in TCGA dataset. Due to the lack of *COL11A1* and *INHBA* information in The Human Protein Atlas dataset, the protein expression level was not evaluated (Figure 4). The results also supported that *SPP1*, *COL11A1*, *ADAM12*, and *INHBA* expressions were significantly higher in colon cancer tissues compared to that of the normal tissues in accordance with our previous study.

## 4 DISCUSSION

In this study, we performed several bioinformatics analyses to excavate key genes involved in development of colon cancer. At first, 109 dysregulated genes were found through the comparison between the normal colon tissues and the colon cancer tissues. Protein–protein network analysis was followed to study the interactions between differently expressed genes and also cluster was studied in succession. Then, we performed survival analysis on those key genes to search the prognostic value genes. Interestingly, we found a total of six genes which are *SPP1*, *VIP*, *COL11A*, *CA2*, *ADAM12*, *INHBA*. We go a step further to validate them both in RNA expression level and protein expression level, also, the results are in accordance with our pervious study.

*SPP1*, a secreted phosphoprotein which contains RGD domain, was firstly separated from bone matrix as an extracellular matrix protein by Herring (Heinegård, Hultenby, Oldberg, Reinholt, & Wendel, 1989; Oldberg, Franzén, & Heinegård, 1986). It is vital in bone reconstruction, anti‐inflammation, arteriosclerosis, and immunomodulation. A variety of cell types including osteoclast, macrophage, epithelial cells, T cells, endothelial cells could secrete *SPP1* (Saitoh, Kuratsu, Takeshima, Yamamoto, & Ushio, 1995). Besides, many studies hold the opinion that *SPP1* participates in the development and metastasis of malignant tumor. In gastric cancer (Imano et al., 2009), esophageal cancer (Lin et al., 2015), glioma (Ellert‐Miklaszewska et al., 2016), breast cancer (Rodrigues, Teixeira, Schmitt, Paulsson, & Lindmark‐Mänsson, 2007), lung cancer (Chambers et al., 1996). It was upregulated and might have served as a biomarker. Also, it could promote ovarian cancer proliferation, migration and invasion in vitro by activating Integrin β1/FAK/AKT signaling pathway (Zeng, Zhou, Wu, & Xiong, 2018). Also, the recent study showed that *SPP1* could mediate macrophage polarization and lung cancer evasion, which could be used as a promising drug target (Zhang, Du, Chen, & Xiang, 2017).

*COL11A1* encodes one of the two alpha chains of type XI collagen, a minor fibrillar collagen. It has been widely studied in many cancers. It is overexpressed in both adenocarcinoma and squamous cell lung carcinoma, comparing with the corresponding non‐neoplastic lung tissues (Wang et al., 2002), in metastatic oral cavity/pharynx squamous cell carcinoma (Schmalbach et al., 2004). Also, it was involved in lymph node metastasis in breast cancer (Feng et al., 2007) which could be used as a potential biomarker to distinguish malignant from premalignant lesions in stomach and pancreas cancer (Kleinert et al., 2015; Zhao et al., 2009). It is localized in the Golgi apparatus of normal human colon goblet cells (Bowen et al., 2008). *COL11A1* may be associated with the APC/beta‐catenin pathway in FAP and sporadic colon cancer (Fischer et al., 2001). In lung cancer, it has a positive correlation with pathology stage, poor prognosis, and lymph node metastasis (Chong et al., 2006), it promotes ovarian cancer progression and chemoresistance to cisplatin and paclitaxel via activating *NF‐KB*, and it promotes the expression of *TWIST1*, *MCL1(*Wu, Huang, Chang, & Chou, 2017). It has also been reported that *COL11A1* was upregulated in gastric cancer and non‐small cell lung cancer which could boost the malignant behavior in vitro (Li, Li, Lin, Zhuo, & Si, 2017; Shen et al., 2016). *COL11A1* could be utilized as a promising biomarker in predicting malignant relapse of breast intraductal papilloma (Freire et al., 2015).

Carbonic anhydrase (CA) II is a member of carbonic anhydrases, which are a ubiquitous group of zinc‐bound metalloenzymes and catalyze the reversible hydration of carbon dioxide. Carbonic anhydrase II (hCAII) has important function in physiology and pathology process. *CA II* highly expresses in different normal organs, but its expression is inhibited in cancer cells (Li, Xie et al., 2012; Sheng, Dong, Zhou, Li, & Dong, 2013). *CA II* is also associated with osteopetrosis and renal tubular acidosis (Borthwick et al., 2003). *CA II* takes part in keeping the adequate balance between carbon dioxide and bicarbonate and controls the pH level in cells. As we know that the carbon dioxide and bicarbonate balance is the basic life activities, and can influence various cell behaviors, the low expression of *CA II* may play important roles in tumor progress and development.

*ADAM12* (ADisintegrin and metalloproteinase domain‐containing protein 12) encodes a member of a family of proteins, which play important role in a variety of biological processes involving cell‐cell and cell‐matrix interactions (Roy, Wewer, Zurakowski, Pories, & Moses, 2004). *ADAM12* have different isoform, that shorter isoforms are secreted, while longer isoforms are membrane‐bound form. *AMDAM12* takes part in the regulation in physic and pathological progress, including muscle development, neurogenesis, and fertilization. *ADAM12* is upregulated in various cancer, including breast, prostate, ovarian, skin, stomach, lung and brain cancers (Li, Duhachek‐Muggy et al., 2012; Shao et al., 2014). *ADAM12* contributes to tumor progression and metastasis by promoting tumor cell proliferation, migration, invasion, and apoptosis resistance.

*INHBA* is a member of the TGF‐beta (transforming growth factor‐beta) superfamily. *INHBA* gene is overexpressed in different kinds of cancer, such as colorectal cancer, pancreatic cancer, and lung cancer, and promotes cell proliferation, invasion, metastasis and chemoresistance in cancer cells (Okano et al., 2013; Oshima et al., 2014). *INHBA* also takes part in the development of eye, tooth and testis. *INHBA* can form different kind of protein complex, which can activate and inhibit follicle stimulating hormone secretion from the pituitary gland, respectively.

In conclusion, in this study, we performed integrated analysis to discover the differently expressed genes involved in the development of colon cancer, also showed a panel of genes with prognostic values to better evaluate the outcome of colon cancer patients. Here, we found that *SPP1*, *VIP*, *COL11A1*, *CA2*, *ADAM12*, and *INHBA* exhibited some significant prognostic values. More in‐depth studies are needed to determine the biological functions and mechanisms through which these genes impact cancer malignant cell behavior. Also, the expression pattern of these genes may be a promising target for therapy in colon cancer.

## CONFLICT OF INTEREST

None declared.

## Supporting information

FigS1

## References

[mgg3588-bib-0001] Borthwick, K. J. , Kandemir, N. , Topaloglu, R. , Kornak, U. , Bakkaloglu, A. , Yordam, N. , … Karet, F. E. (2003). A phenocopy of CAII deficiency: A novel genetic explanation for inherited infantile osteopetrosis with distal renal tubular acidosis. Journal of Medical Genetics, 40(2), 115–121. 10.1136/jmg.40.2.115 12566520 PMC1735376

[mgg3588-bib-0002] Bowen, K. B. , Reimers, A. P. , Luman, S. , Kronz, J. D. , Fyffe, W. E. , & Oxford, J. T. (2008). Immunohistochemical localization of collagen type XI alpha1 and alpha2 chains in human colon tissue. Journal of Histochemistry and Cytochemistry, 56(3), 275–283. 10.1369/jhc.7a7310.2007 18040076 PMC2324180

[mgg3588-bib-0003] Chambers, A. F. , Wilson, S. M. , Kerkvliet, N. , O'Malley, F. P. , Harris, J. F. , & Casson, A. G. (1996). Osteopontin expression in lung cancer. Lung Cancer, 15(3), 311–323. 10.1016/0169-5002(95)00595-1 8959677

[mgg3588-bib-0004] Chandrashekar, D. S. , Bashel, B. , Balasubramanya, S. A. H. , Creighton, C. J. , Rodriguez, I. P. , Chakravarthi, B. V. S. K. , & Varambally, S. (2017). UALCAN: A portal for facilitating tumor subgroup gene expression and survival analyses. Neoplasia, 19(8), 649–658. 10.1016/j.neo.2017.05.002 28732212 PMC5516091

[mgg3588-bib-0005] Chong, I. W. , Chang, M. Y. , Chang, H. C. , Yu, Y. P. , Sheu, C. C. , Tsai, J. R. , … Lin, S. R. (2006). Great potential of a panel of multiple hMTH1, SPD, ITGA11 and COL11A1 markers for diagnosis of patients with non‐small cell lung cancer. Oncology Reports, 16(5), 981–988. 17016581

[mgg3588-bib-0006] Ellert‐Miklaszewska, A. , Wisniewski, P. , Kijewska, M. , Gajdanowicz, P. , Pszczolkowska, D. , Przanowski, P. , … Kaminska, B. (2016). Tumour‐processed osteopontin and lactadherin drive the protumorigenic reprogramming of microglia and glioma progression. Oncogene, 35(50), 6366–6377. 10.1038/onc.2016.55 27041573

[mgg3588-bib-0007] Feng, Y. , Sun, B. , Li, X. , Zhang, L. , Niu, Y. , Xiao, C. , … Hao, X. (2007). Differentially expressed genes between primary cancer and paired lymph node metastases predict clinical outcome of node‐positive breast cancer patients. Breast Cancer Research and Treatment, 103(3), 319–329. 10.1007/s10549-006-9385-7 17123152

[mgg3588-bib-0008] Fischer, H. , Salahshor, S. , Stenling, R. , Björk, J. , Lindmark, G. , Iselius, L. , … Lindblom, A. (2001). COL11A1 in FAP polyps and in sporadic colorectal tumors. BMC Cancer, 1, 17 10.1186/1471-2407-1-17 11707154 PMC59693

[mgg3588-bib-0009] Freire, J. , García‐Berbel, L. , García‐Berbel, P. , Pereda, S. , Azueta, A. , García‐Arranz, P. , … Gómez‐Román, J. (2015). Collagen type XI alpha 1 expression in intraductal papillomas predicts malignant recurrence. BioMed Research International, 2015, 812027 10.1155/2015/812027 26448946 PMC4584034

[mgg3588-bib-0010] Haggar, F. A. , & Boushey, R. P. (2009). Colorectal cancer epidemiology: Incidence, mortality, survival, and risk factors. Clinics in Colon and Rectal Surgery, 22(4), 191–197. 10.1055/s-0029-1242458 21037809 PMC2796096

[mgg3588-bib-0011] He, J. , Pei, L. , Jiang, H. , Yang, W. , Chen, J. , & Liang, H. (2017). Chemoresistance of colorectal cancer to 5‐fluorouracil is associated with silencing of the BNIP3 gene through aberrant methylation. Journal of Cancer, 8(7), 1187–1196. 10.7150/jca.18171 28607593 PMC5463433

[mgg3588-bib-0012] Heinegård, D. , Hultenby, K. , Oldberg, A. , Reinholt, F. , & Wendel, M. (1989). Macromolecules in bone matrix. Connective Tissue Research, 21(1–4), 3–11; discussion 12–4. 10.3109/03008208909049990 2691197

[mgg3588-bib-0013] Imano, M. , Satou, T. , Itoh, T. , Sakai, K. , Ishimaru, E. , Yasuda, A. , … Ohyanagi, H. (2009). Immunohistochemical expression of osteopontin in gastric cancer. Journal of Gastrointestinal Surgery, 13(9), 1577–1582. 10.1007/s11605-009-0955-y 19582521

[mgg3588-bib-0014] Kamps, R. , Brandão, R. D. , van den Bosch, B. J. , Paulussen, A. D. C. , Xanthoulea, S. , Blok, M. J. , & Romano, A. (2017). Next‐generation sequencing in oncology: Genetic diagnosis, risk prediction and cancer classification. International Journal of Molecular Sciences, 18(2), 308 10.3390/ijms18020308 PMC5343844 28146134

[mgg3588-bib-0015] Kleinert, R. , Prenzel, K. , Stoecklein, N. , Alakus, H. , Bollschweiler, E. , Hölscher, A. , & Warnecke‐Eberz, U. (2015). Gene expression of Col11A1 is a marker not only for pancreas carcinoma but also for adenocarcinoma of the papilla of vater, discriminating between carcinoma and chronic pancreatitis. Anticancer Research, 35(11), 6153–6158. 26504042

[mgg3588-bib-0016] Li, H. , Duhachek‐Muggy, S. , Qi, Y. , Hong, Y. , Behbod, F. , & Zolkiewska, A. (2012). An essential role of metalloprotease‐disintegrin ADAM12 in triple‐negative breast cancer. Breast Cancer Research and Treatment, 135(3), 759–769. 10.1007/s10549-012-2220-4 22926263 PMC3470813

[mgg3588-bib-0017] Li, A. , Li, J. , Lin, J. , Zhuo, W. , & Si, J. (2017). COL11A1 is overexpressed in gastric cancer tissues and regulates proliferation, migration and invasion of HGC‐27 gastric cancer cells in vitro. Oncology Reports, 37(1), 333–340. 10.3892/or.2016.5276 28004111

[mgg3588-bib-0018] Li, X. J. , Xie, H. L. , Lei, S. J. , Cao, H. Q. , Meng, T. Y. , & Hu, Y. L. (2012). Reduction of CAII expression in gastric cancer: Correlation with invasion and metastasis. Chinese Journal of Cancer Research, 24(3), 196–200. 10.1007/s11670-012-0196-6 23359292 PMC3555285

[mgg3588-bib-0019] Lin, J. , Myers, A. L. , Wang, Z. , Nancarrow, D. J. , Ferrer‐Torres, D. , Handlogten, A. , … Lin, L. (2015). Osteopontin (OPN/SPP1) isoforms collectively enhance tumor cell invasion and dissemination in esophageal adenocarcinoma. Oncotarget, 6(26), 22239–22257. 26068949 10.18632/oncotarget.4161 PMC4673160

[mgg3588-bib-0020] Marin, J. J. , Sanchez de Medina, F. , Castaño, B. , Bujanda, L. , Romero, M. R. , Martinez‐Augustin, O. , …, Briz, O. (2012). Chemoprevention, chemotherapy, and chemoresistance in colorectal cancer. Drug Metabolism Reviews, 44(2), 148–172. 10.3109/03602532.2011.638303 22497631

[mgg3588-bib-0021] Okano, M. , Yamamoto, H. , Ohkuma, H. , Kano, Y. , Kim, H. , Nishikawa, S. , … Ishii, H. (2013). Significance of INHBA expression in human colorectal cancer. Oncology Reports, 30(6), 2903–2908. 10.3892/or.2013.2761 24085226

[mgg3588-bib-0022] Oldberg, A. , Franzén, A. , & Heinegård, D. (1986). Cloning and sequence analysis of rat bone sialoprotein (osteopontin) cDNA reveals an Arg‐Gly‐Asp cell‐binding sequence. Proceedings of the National Academy of Sciences of the United States of America, 83(23), 8819–8823. 10.1073/pnas.83.23.8819 3024151 PMC387024

[mgg3588-bib-0023] Oshima, T. , Yoshihara, K. , Aoyama, T. , Hasegawa, S. , Sato, T. , Yamamoto, N. , … Masuda, M. (2014). Relation of INHBA gene expression to outcomes in gastric cancer after curative surgery. Anticancer Research, 34(5), 2303–2309. 24778035

[mgg3588-bib-0024] Robinson, M. D. , McCarthy, D. J. , & Smyth, G. K. (2010). edgeR: A Bioconductor package for differential expression analysis of digital gene expression data. Bioinformatics, 26(1), 139–140. 10.1093/bioinformatics/btp616 19910308 PMC2796818

[mgg3588-bib-0025] Rodrigues, L. R. , Teixeira, J. A. , Schmitt, F. L. , Paulsson, M. , & Lindmark‐Mänsson, H. (2007). The role of osteopontin in tumor progression and metastasis in breast cancer. Cancer Epidemiology, Biomarkers & Prevention, 16(6), 1087–1097. 10.1158/1055-9965.EPI-06-1008 17548669

[mgg3588-bib-0026] Roy, R. , Wewer, U. M. , Zurakowski, D. , Pories, S. E. , & Moses, M. A. (2004). ADAM 12 cleaves extracellular matrix proteins and correlates with cancer status and stage. Journal of Biological Chemistry, 279(49), 51323–51330. 15381692 10.1074/jbc.M409565200

[mgg3588-bib-0027] Saitoh, Y. , Kuratsu, J. , Takeshima, H. , Yamamoto, S. , & Ushio, Y. (1995). Expression of osteopontin in human glioma. Laboratory Investigation, 72(1), 55–63. 7837791

[mgg3588-bib-0028] Schmalbach, C. E. , Chepeha, D. B. , Giordano, T. J. , Rubin, M. A. , Teknos, T. N. , Bradford, C. R. , … Hanash, S. (2004). Molecular profiling and the identification of genes associated with metastatic oral cavity/pharynx squamous cell carcinoma. Archives of Otolaryngology ‐ Head and Neck Surgery, 130(3), 295–302. 15023835 10.1001/archotol.130.3.295

[mgg3588-bib-0029] Shannon, P. , Markiel, A. , Ozier, O. , Baliga, N. S. , Wang, J. T. , Ramage, D. , … Ideker, T. (2003). Cytoscape: A software environment for integrated models of biomolecular interaction networks. Genome Research, 13(11), 2498–2504. 10.1101/gr.1239303 14597658 PMC403769

[mgg3588-bib-0030] Shao, S. , Li, Z. , Gao, W. , Yu, G. , Liu, D. , & Pan, F. (2014). ADAM‐12 as a diagnostic marker for the proliferation, migration and invasion in patients with small cell lung cancer. PLoS ONE, 9(1), e85936 10.1371/journal.pone.0085936 24465799 PMC3897605

[mgg3588-bib-0031] Shen, L. , Yang, M. , Lin, Q. , Zhang, Z. , Zhu, B. , & Miao, C. (2016). COL11A1 is overexpressed in recurrent non‐small cell lung cancer and promotes cell proliferation, migration, invasion and drug resistance. Oncology Reports, 36(2), 877–885. 10.3892/or.2016.4869 27373316

[mgg3588-bib-0032] Sheng, W. , Dong, M. , Zhou, J. , Li, X. , & Dong, Q. (2013). Down regulation of CAII is associated with tumor differentiation and poor prognosis in patients with pancreatic cancer. Journal of Surgical Oncology, 107(5), 536–543. 10.1002/jso.23282 23090763

[mgg3588-bib-0033] Szklarczyk, D. , Morris, J. H. , Cook, H. , Kuhn, M. , Wyder, S. , Simonovic, M. , … von Mering, C. (2017). The STRING database in 2017: Quality‐controlled protein–protein association networks, made broadly accessible. Nucleic Acids Research, 45(D1): D362–D368. 10.1093/nar/gkw937 27924014 PMC5210637

[mgg3588-bib-0034] Tang, Z. , Li, C. , Kang, B. , Gao, G. , Li, C. , & Zhang, Z. (2017). GEPIA: A web server for cancer and normal gene expression profiling and interactive analyses. Nucleic Acids Research, 45(W1), W98–W102. 10.1093/nar/gkx247 28407145 PMC5570223

[mgg3588-bib-0035] Uhlén, M. , Fagerberg, L. , Hallström, B. M. , Lindskog, C. , Oksvold, P. , Mardinoglu, A. , … Pontén, F. (2015). Proteomics. Tissue‐based map of the human proteome. Science, 347(6220), 1260419 10.1126/science.1260419 25613900

[mgg3588-bib-0036] Wang, K. K. , Liu, N. , Radulovich, N. , Wigle, D. A. , Johnston, M. R. , Shepherd, F. A. , … Tsao, M. S. (2002). Novel candidate tumor marker genes for lung adenocarcinoma. Oncogene, 21(49), 7598–7604. 10.1038/sj.onc.1205953 12386823

[mgg3588-bib-0037] Wu, Y. H. , Huang, Y. F. , Chang, T. H. , & Chou, C. Y. (2017). Activation of TWIST1 by COL11A1 promotes chemoresistance and inhibits apoptosis in ovarian cancer cells by modulating NF‐κB‐mediated IKKβ expression. International Journal of Cancer, 141(11), 2305–2317. 10.1002/ijc.30932 28815582

[mgg3588-bib-0038] Yu, G. , Wang, L. G. , Han, Y. , & He, Q. Y. (2012). clusterProfiler: An R package for comparing biological themes among gene clusters. OMICS: A Journal of Integrative Biology, 16(5), 284–287. 10.1089/omi.2011.0118 22455463 PMC3339379

[mgg3588-bib-0039] Zeng, B. , Zhou, M. , Wu, H. , & Xiong, Z. (2018). SPP1 promotes ovarian cancer progression via Integrin β1/FAK/AKT signaling pathway. OncoTargets and Therapy, 12(11), 1333–1343. 10.2147/ott.s154215 PMC5856063 29559792

[mgg3588-bib-0040] Zhang, Y. , Du, W. , Chen, Z. , & Xiang, C. (2017). Upregulation of PD‐L1 by SPP1 mediates macrophage polarization and facilitates immune escape in lung adenocarcinoma. Experimental Cell Research, 359(2), 449–457. 10.1016/j.yexcr.2017.08.028 28830685

[mgg3588-bib-0041] Zhao, Y. , Zhou, T. , Li, A. , Yao, H. , He, F. , Wang, L. , & Si, J. (2009). A potential role of collagens expression in distinguishing between premalignant and malignant lesions in stomach. The Anatomical Record, 292(5), 692–700. 10.1002/ar.20874 19306436

