# Supplementary figures and images for: Integrated bioinformatics analysis of key genes involved in progress of colon cancer
Source: Mol Genet Genomic Med. 2019 Feb 11;7(4):e00588. doi: 10.1002/mgg3.588 (PMC6465657; doi:10.1002/mgg3.588)

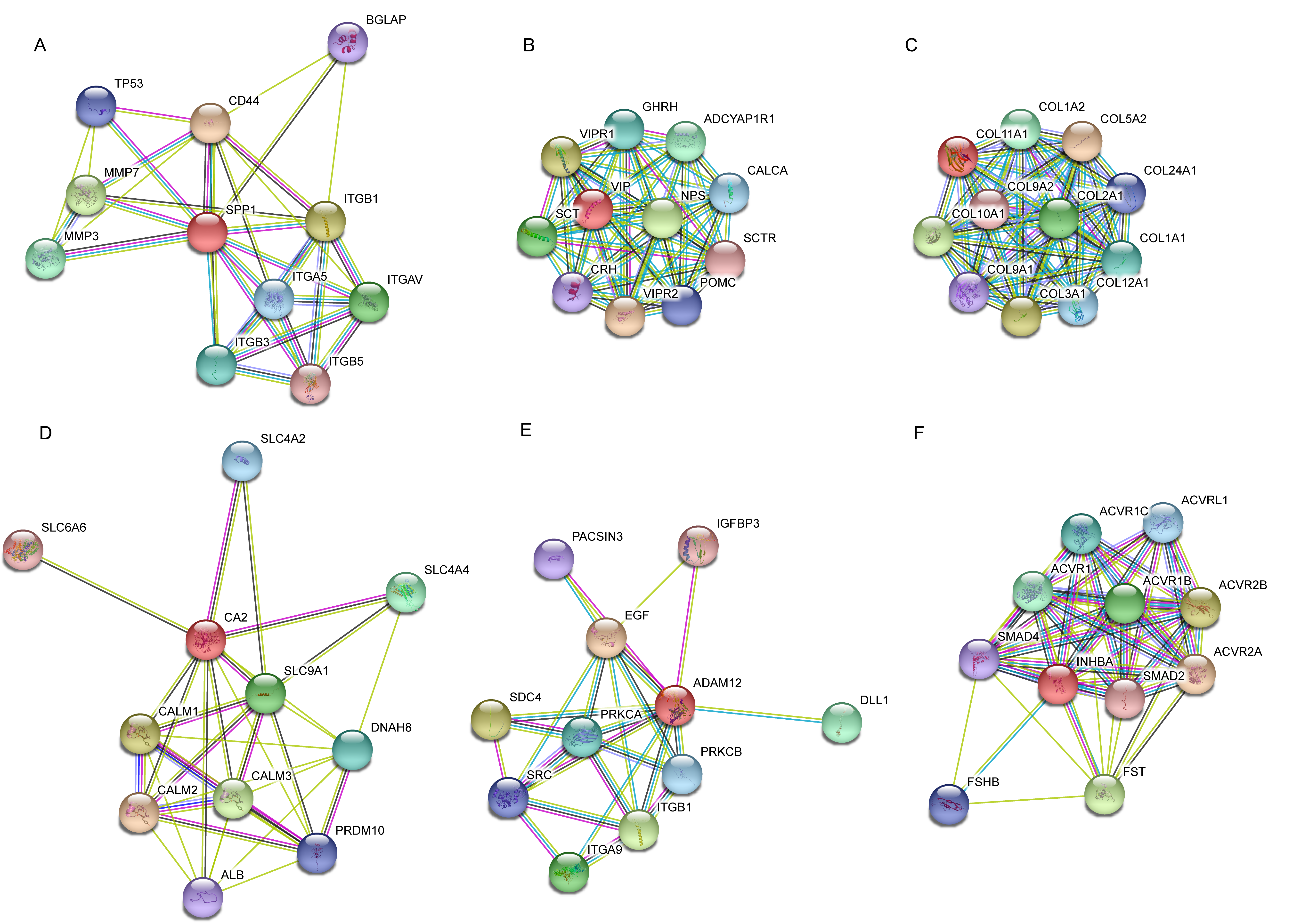

Supplement: Supplementary file 1 — FigS1 [file MGG3-7-na-s001.tif]
